# Effects of Thyme (*Thymus vulgaris* L.) Essential Oil on Aging-Induced Brain Inflammation and Blood Telomere Attrition in Chronologically Aged C57BL/6J Mice

**DOI:** 10.3390/antiox12061178

**Published:** 2023-05-30

**Authors:** Dwina Juliana Warman, Huijuan Jia, Hisanori Kato

**Affiliations:** 1 Health Nutrition, Department of Applied Biological Chemistry, Graduate School of Agricultural and Life Sciences, The University of Tokyo, 1-1-1 Yayoi, Bunkyo-ku, Tokyo 113-8657, Japan; warman-dwina-juliana681@g.ecc.u-tokyo.ac.jp; 2 Department of Applied Nutrition, School of Nutrition, Kagawa Nutrition University, 3-9-21 Chiyoda, Sakado-shi 350-0288, Japan

**Keywords:** inflammaging, chronological aging, telomere shortening, thyme essential oil, thymol, p-cymene, antioxidant, anti-inflammation, healthy aging

## Abstract

Chronological aging is commonly accompanied by chronic low-grade inflammation (or “inflammaging”), a contributor to the development of age-related chronic diseases. Aging increases oxidative stress that accelerates telomere shortening, leading to cell senescence and the generation of senescence-associated secretory phenotype (SASP) that exacerbates inflammation. Dietary antioxidants may help protect telomeres and attenuate inflammation. Thyme essential oil (TEO), reported for its potency against neuroinflammation, was fed to chronologically aged C57BL/6J mice for 24 weeks. The TEO diet showed notable impacts on the hippocampus, indicated by lower expression of the aging-related gene *p16^INK4A^* (*p* = 0.0783) and significantly lower expression of cyclin D kinase *Cdk4* and *Cdk6* (*p* < 0.05) compared to the age-matched control mice. The TEO group also showed significantly lower gene expression of the pro-inflammatory cytokine *Il6* (*p* < 0.05) in the hippocampus and lower *Il1b* expression in the liver and cerebellum (*p* < 0.05). In vitro experiments conducted on NIH-3T3 cells expressing SASP revealed the dose-dependent anti-inflammatory activity of TEO. Remarkably, TEO diet-fed mice showed higher survival rates and significantly longer blood telomere lengths than the control mice. Monoterpene antioxidants in TEO, particularly thymol and p-cymene, may primarily contribute to the anti-inflammatory and telomere-protecting activities of TEO.

## 1. Introduction

Aging is attributed to the finite proliferative capacity of mammalian cells, known as the “Hayflick limit”, which leads to cell senescence [1]. The accumulation of senescent cells triggers the senescence-associated secretory phenotype (SASP), which exacerbates the inflammatory response that follows the aging process [2]. Aging-induced inflammation is known as chronic low-grade inflammation or inflammaging. Interestingly, this chronic inflammation was indicated by increasing serum levels of interleukin (IL)-6, IL-1β, and tumor necrosis factor-α (TNF-α), even in healthy elderly humans [3,4,5,6] and aged mice [7,8]. For instance, Schadel et al. reported that pro-inflammatory cytokines, including IL-6, IL-1β, and TNF-α, and chemokines, particularly C-C motif chemokine ligand 2 (CCL2) and C-X-C motif chemokine ligand 1 (CXCL1), were highly expressed at the protein level in a tissue-dependent manner in biologically aged C57BL/6 mice [7]. Increased expression of *Il1b*, *Tnfa*, and *Ccl2* was also observed at the mRNA level [8]. Chronic low-grade inflammation accompanying aging is suggested to be a major contributor to age-related chronic diseases, including cancer, dementia, and stroke [9,10,11], which consequently reduces the quality of life at a later age.

Cell aging in the brain also promotes neuroinflammation because of deterioration in the brain’s ability to balance pro- and anti-inflammatory cytokines, eventually shifting to the pro-inflammatory state [12]. Under normal physiological conditions, the gene encoding IL-6 in the brain is relatively low [13], but Ye and Johnson reported that IL-6 secretion increases in the hippocampus, cerebral cortex, and cerebellum of aged mice [14]. A higher release of IL-6 in the aged brain may be sufficient to predispose an individual to the onset of neurodegenerative disease (ND) [14]. Neuroinflammation may also trigger the development of age-related NDs, including Alzheimer’s and Parkinson’s disease [15], the two most common NDs in the elderly [16]. Therefore, attenuating inflammation in the brain during aging may be significant in supporting healthy aging.

Thyme (*Thymus vulgaris* L.), a type of herb originating from the Mediterranean region, has gained interest after recent research revealed that its bioactive compounds might be beneficial in protecting the brain from ND risk factors and neuroinflammation. Shimada et al. first reported the glutaminase inhibitory activities of three pentacyclic terpenes isolated from *Thymus vulgaris* L.: ursolic acid, betulinic acid, and oleanolic acid [17]. Glutaminase inhibitors are potential therapeutic agents to prevent and treat NDs [17]. Another recent study successfully elucidated the anti-inflammatory activity of linalool and geraniol in thyme essential oil (TEO) against neuroinflammation in lipopolysaccharide-induced BV-2 microglial cells [18]. Furthermore, thymol and p-cymene, either alone or in combination, attenuated cholinergic affliction, which contributes to NDs [19]. However, the effects of TEO against chronic low-grade inflammation in spontaneous aging have not yet been investigated.

This study offers a new approach to prevent the detrimental effects of aging-associated chronic low-grade inflammation via diet intervention by incorporating TEO into the mouse diet. To the best of our knowledge, this study is the first to investigate the effects of TEO on inflammation in various parts of the brain (hippocampus, cerebellum, and cerebral cortex) and on blood telomere length in chronologically aged mice. Telomere shortening, a hallmark of aging, is a major contributor to replicative senescence through DNA damage response [20], which in turn activates cell cycle arrest [21]. Aging increases oxidative stress, which accelerates telomere attrition [22]. Protection of telomeres along with alleviation of brain inflammation through dietary TEO supplementation is expected to promote healthy aging and increase global healthy life expectancy.

## 2. Materials and Methods

### 2.1. Gas Chromatography-Mass Spectrometry (GC/MS)

TEO was obtained from Inabata Koryo Co., Ltd. (Osaka, Japan). The supplier analyzed the chemical composition of the oil using an HP5890A gas chromatograph (Agilent Technologies, Tokyo, Japan) equipped with a split/splitless injector (split ratio 100:1) and a flame ionization detector, and connected to an HP5973 mass spectrometer (Agilent Technologies, Tokyo, Japan). A DB-WAX column with a diameter of 0.25 mm and a length of 30 m was used. The temperature was programmed to increase from 50 °C to 240 °C at 3 °C/min. The composition of the volatile compounds is presented in Table 1.

### 2.2. Diets

Previous studies revealed that a TEO dosage of 250 mg/kg body weight (BW)/day, equal to 0.2% (*w/w*), started to exert significant anti-inflammatory effects in mice [23,24]. This dose does not exceed the oral LD50 value of TEO determined for mice (4000 mg/kg BW/day) [23]. We confirmed that supplementation of TEO at 0.2% (*w/w*) into the mouse diet did not affect food intake and BW in our preliminary research; therefore, it was used in this research. TEO diet formulation was based on the control diet AIN-93G, the composition of which is presented in Table S1.

### 2.3. Animal Breeding and Grouping

All animal experiments in this study were approved by the Animal Care and Use Committee of The University of Tokyo (P21-100 and 2021/10/15). Male C57BL/6J mice that were 8- and 53-week-old (wo) were purchased from Oriental Yeast Co., Ltd. (Tokyo, Japan) and were divided into three groups: Young control (Young, 8-wo, *n* = 10), Old control (CON, 53-wo, *n* = 10), and TEO (53-wo, *n* = 11). They were housed in groups under controlled conditions (50 ± 10% relative humidity, 23 ± 2 °C, and a 12/12-h light/dark cycle). All groups were fed the control AIN-93 diet for five days for acclimation, after which the diets were switched as follows. The Young and CON groups were fed the AIN-93 diet, whereas the TEO group was fed the diet containing 0.2% TEO for 24 weeks. Mice were provided with food and water *ad libitum*. Body weight was measured once per week, while food intake was recorded twice per week.

### 2.4. Blood Collection and Tissue Harvesting

All mice were euthanized after 24 weeks. Before euthanasia, the mice were anesthetized with isoflurane (MSD, Kenilworth, NJ, USA). Blood samples were collected from the inferior vena cava. After centrifugation at 3000 rpm for 15 min at 4 °C, the blood plasma was isolated and stored at −80 °C for further biochemical analysis. The brain (hippocampus, cerebellum, and cerebral cortex), liver, spleen, kidney, gastrocnemius muscle, soleus muscle, and adipose tissues (retroperitoneal, mesenteric, and epididymal fat) were collected manually. The brain sampling regions are shown in Figure S1. For RNA extraction, tissue samples from the hippocampus, cerebellum, cerebral cortex, and liver were submerged individually in 2 mL tubes containing 1 mL Invitrogen RNAlater Stabilization Solution (Thermo Fisher Scientific, Tokyo, Japan) and stored at −80 °C until analysis. The remaining tissues were immediately frozen in liquid nitrogen after harvesting and subsequently stored at −80 °C for further analysis.

### 2.5. DNA Extraction

Before extracting blood DNA from the blood lymphocytes, the red blood cells were removed using a gradient density medium, Lympholyte-M CL5030 (Cederlane Labs, Burlington, ON, Canada), following the manufacturer’s instructions. The total DNA was extracted using Qiagen DNeasy Blood and Tissue Kit (Cat. no. 69504; Tokyo, Japan), following the manufacturer’s protocol. The DNA quality was determined using NanoDrop™ One/OneC (Thermo Fisher Scientific, Tokyo, Japan), where a 260/280 ratio of >1.8 and a 260/230 ratio between 2.0–2.2 were considered pure DNA.

### 2.6. Absolute Telomere Length Quantification

Absolute telomere length was measured from DNA extracted from the blood (as described above) using the Absolute Mouse Telomere Length Quantification qPCR Assay Kit (Cat no. M8918; ScienCell, Carlsbad, CA, USA), following the kit protocol.

### 2.7. Cell Culture Experiment

The mouse embryonic fibroblast NIH/3T3 cell line was used as a model to observe the SASP phenomenon in cell culture. It was purchased from the Japanese Collection of Research Bioresources (JCRB) Cell Bank (JCRB0615). The cells were cultured in Dulbecco’s Modified Eagle’s Medium (DMEM, #D6046; Sigma-Aldrich, Burlington, MA, USA) containing 10% newborn calf serum (NCS, #N4762; Sigma Aldrich) and 1% penicillin/streptomycin (PS, #P0781; Sigma-Aldrich). Cell culturing was performed on 10 mm Petri dishes that were incubated at 37 °C with 5% CO_2_. The medium was changed every other day, and subculturing was performed when the cell confluency reached 80–85%.

To establish a SASP model, mitomycin C (MMC, #M4287; Sigma Aldrich) was used to induce DNA damage and, subsequently, cell senescence. NIH/3T3 cells (1 × 10^5^ cells/well, 2 mL/well) were grown in a six-well plate in a normal medium. After 24 h incubation, the MMC group was treated with 0.3 μM MMC for 24 h at 37 °C, following which the medium was changed to DMEM medium and cultured for another 24 h.

TEO experiments were conducted as follows. NIH/3T3 cells at a density of 1 × 10^5^ cells/well were grown in a six-well plate (2 mL/well) in a normal medium for 24 h. They were then divided into five groups (*n* = 3 per group): Con (control), MMC, 30 μg/mL TEO, 60 μg/mL TEO, and 120 μg/mL TEO. For TEO pretreatment, the Con and MMC groups were cultured in a normal medium, while the TEO groups were treated with 30, 60, or 120 μg/mL of TEO for 24 h. After incubation, the Con group medium was exchanged with the normal medium, while the media of MMC and all TEO groups were exchanged with 0.3 μM MMC. The cells were cultured for 24 h at 37 °C, and then the media were changed to a normal medium for another 24 h of incubation. For TEO pre- and co-treatment, the TEO groups were treated with 30, 60, or 120 μg/mL of TEO for 24 h, then followed by treatment with both 0.3 μM MMC and 30, 60, or 120 μg/mL of TEO for 24 h at 37 °C. Finally, the media were changed to a normal medium for another 24 h of incubation. The cells were harvested with a lysis buffer from Nucleospin RNA Plus (Macherey-Nagel, Duren, Germany), and RNA was extracted following the manufacturer’s instructions.

Senescence-associated beta-galactosidase (SA-β-Gal) senescence-based assay was performed by using the Cellular Senescence Detection Kit (#CBA-230; Cell Biolabs, Tokyo, Japan), following the manufacturer’s protocol.

### 2.8. RNA Extraction

Total RNA from the hippocampus, cerebellum, and cerebral cortex was extracted using Invitrogen TRIzol Reagent (Carlsbad, CA, USA), whereas RNA from the liver and cell culture was extracted using NucleoSpin RNA Plus (Macherey-Nagel), following the manufacturer’s protocols. RNA quality was determined using NanoDrop™ One/OneC (Thermo Fisher Scientific), where a 260/280 ratio of >2.0 and 260/230 ratio between 2.0–2.2 were considered as pure RNA.

### 2.9. Quantitative Real-Time Polymerase Chain Reaction (qRT-PCR)

First, complementary DNA (cDNA) was generated from the extracted total RNA using PrimeScript™ RT Master Mix (Perfect Real Time; Takara Bio Inc.; Shiga, Japan). Real-time PCR and quantification of PCR products were performed on a Thermal Cycler Dice TP800 (Takara Bio Inc., Madison, WI, USA) using SYBR Premix Ex Taq II (Takara Biotechnology Co., Ltd., Shiga, Japan). Primers were designed using Primer3Plus, and their sequences are listed in Table S2. The qPCR thermal cycling conditions were as follows: Denaturation at 95 °C for 30 s, followed by 40 cycles at 95 °C for 5 s, then annealing and extension at 60 °C for 30 s. The relative expression levels of products were normalized to the housekeeping gene β-actin (*Actb*) for the hippocampus, cerebellum, and cerebral cortex. For liver samples, the relative expression was normalized to the housekeeping gene glyceraldehyde-3-phosphate dehydrogenase (*Gapdh*). All samples were analyzed in triplicate.

### 2.10. Statistical Analysis

All values are presented as the mean ± standard error (SE). The data were analyzed using one-way analysis of variance (ANOVA), followed by evaluation of significant differences using Tukey’s HSD test. A *p*-value < 0.05 was considered statistically significant.

## 3. Results

### 3.1. Body Weight and Total Food Intake

Initially, the old mice had significantly heavier body weight, averaging at 34.6 ± 0.4 g, while the young mice were averagely weighed 23.2 ± 0.33 g. The body weight of Young group mice (8-wo upon arrival) remained significantly lower than that of the CON (53-wo upon arrival) and TEO (53-wo upon arrival) mice from weeks 1 to 21 (Figure S2a). At weeks 22–24, the body weight of Young mice did not significantly differ from that of the TEO mice. Further, the TEO diet did not significantly affect the absolute or relative food intake compared to both the Young and CON groups (Figure S2c,d, respectively).

### 3.2. Organ and Tissue Weights

**Figure 1:**
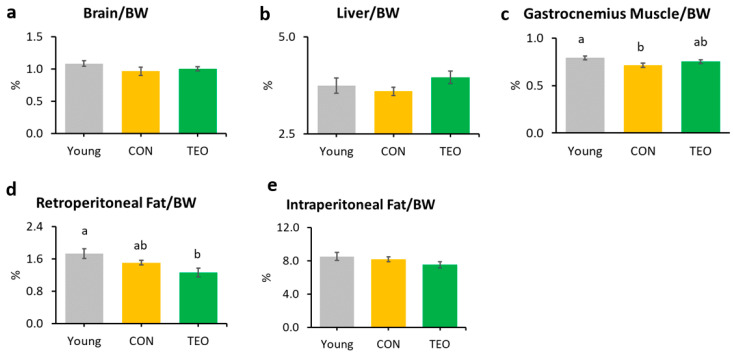
Organ and tissue relative weights per 100 g of body weight (BW) in each experimental group, including the: (**a**) brain; (**b**) liver; (**c**) gastrocnemius muscle; (**d**) retroperitoneal fat; and (**e**) intraperitoneal fat. All values are expressed as the mean ± SE (*n* = 7–11). Different letters indicate significant differences (*p* < 0.05, Tukey’s HSD test).

The gastrocnemius muscle weight in the CON group was significantly lower than that in the Young group (Figure 1c). Notably, the TEO diet significantly reduced retroperitoneal fat compared to the other groups (Figure 1d). There was no significant difference in the relative weights of the brain, liver, kidney, spleen, soleus muscle, and mesenteric fat (Figure 1a,b and Figure S3).

### 3.3. Survival Rate

**Figure 2:**
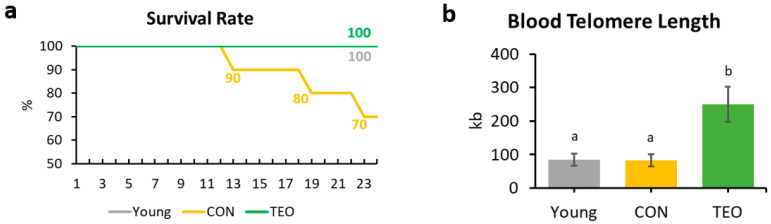
Survival rate and blood telomere length of the mice. (**a**) Mice survival rate within the Young, CON, and TEO groups during the 24-week experimental period; (**b**) Final telomere length measured from blood DNA. All values are expressed as the mean ± SE (*n* = 7–9). kb, kilobases. Different letters indicate significant differences (*p* < 0.05, Tukey’s HSD test).

At the end of the experiment (week 24), the survival rates of the Young, CON, and TEO groups were 100%, 70%, and 100%, respectively (Figure 2a).

### 3.4. Telomere Length

Telomere length was measured from the blood DNA. A significantly longer blood telomere length was observed in the TEO group than in the Young and CON groups (*p* < 0.05, Figure 2b).

### 3.5. Aging-Related and Pro-Inflammatory Cytokine Gene Expression in the Liver, Hippocampus, Cerebellum, and Cerebral Cortex

**Figure 3:**
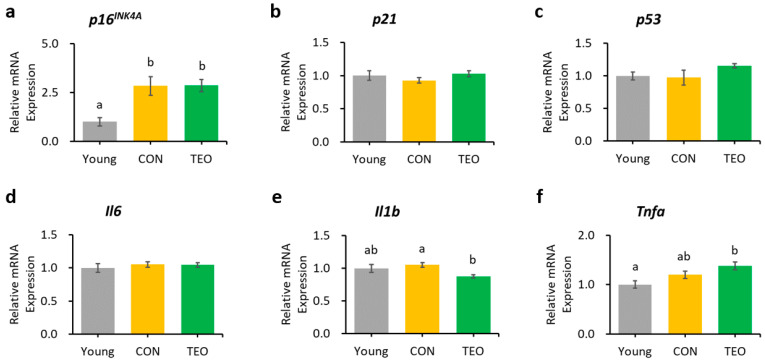
Gene expression in the liver. Aging-related genes: (**a**) *p16^INK4A^*; (**b**) *p21*; and (**c**) *p53*; Pro-inflammatory cytokines: (**d**) *Il6*; (**e**) *Il1b*; and (**f**) *Tnfa*. The above genes were measured using RT-PCR and normalized to the housekeeping gene *Gapdh*. All values are expressed as the mean ± SE (*n* = 6–8). Different letters indicate significant differences (*p* < 0.05, Tukey’s HSD test).

**Figure 4:**
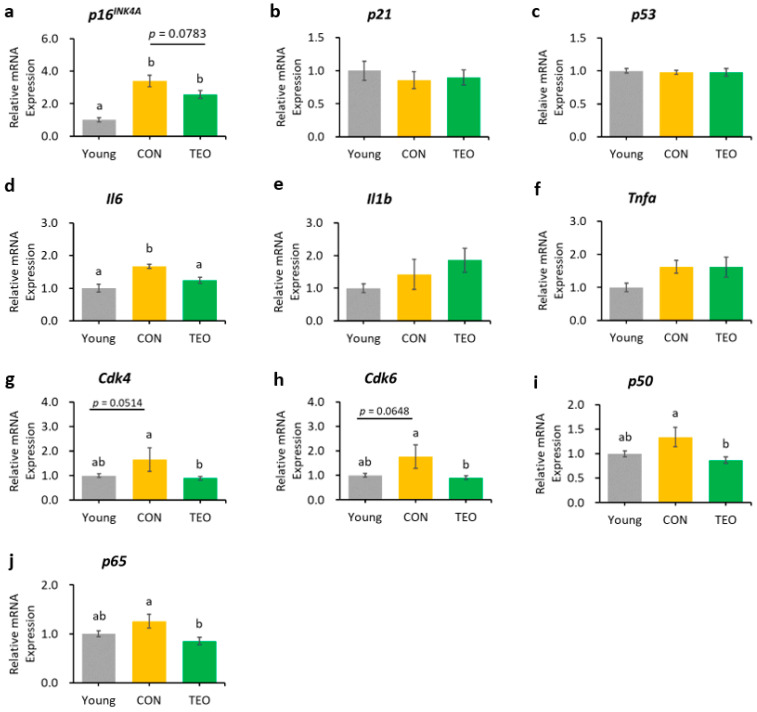
Gene expression in the hippocampus. Aging-related genes: (**a**) *p16^INK4A^*; (**b**) *p21*; and (**c**) *p53*; Pro-inflammatory cytokines: (**d**) *Il6*; (**e**) *Il1b*; and (**f**) *Tnfa*; Downstream molecules in the p16^INK4A^ pathway: (**g**) *Cdk4*; and (**h**) *Cdk6*; NF-κB transcription factors: (**i**) *p65*; and (**j**) *p50*. All genes were measured using RT-PCR and normalized to the housekeeping gene *Actb*. All values are expressed as the mean ± SE (*n* = 6–9). Different letters indicate significant differences (*p* < 0.05, Tukey’s HSD test).

**Figure 5:**
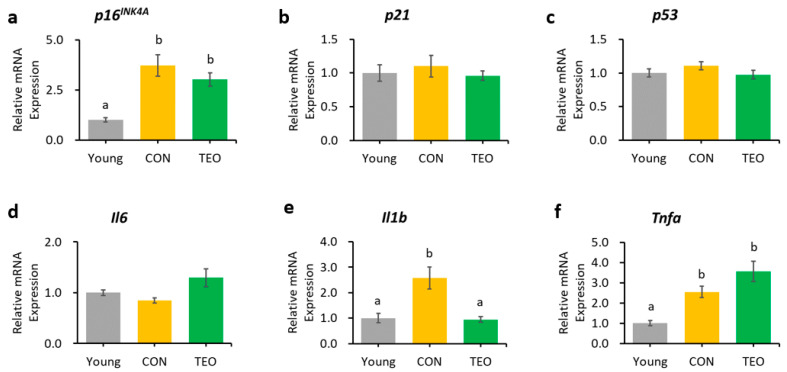
Genes as expressed in the cerebellum. Aging-related genes: (**a**) *p16^INK4A^*; (**b**) *p21*; and (**c**) *p53*; Pro-inflammatory cytokines: (**d**) *Il6*; (**e**) *Il1b*; and (**f**) *Tnfa*. The above genes were measured using RT-PCR and normalized to the housekeeping gene *Actb*. All values are expressed as the mean ± SE (*n* = 6–8). Different letters indicate significant differences (*p* < 0.05, Tukey’s HSD test).

**Figure 6:**
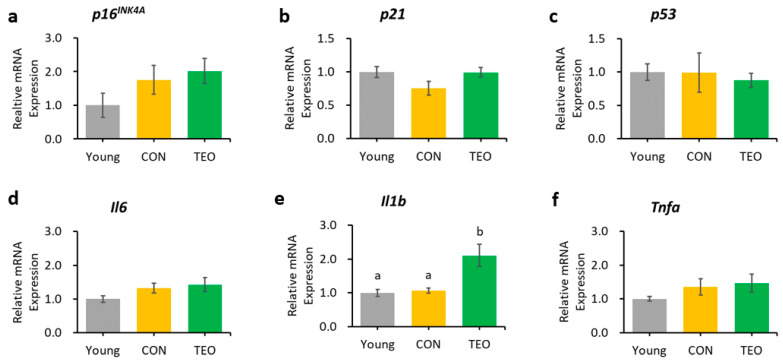
Genes as expressed in the brain’s cerebral cortex. Aging-related genes: (**a**). *p16^INK4A^*; (**b**) *p21*; and (**c**) *p53*; Pro-inflammatory cytokines: (**d**) *Il6*; (**e**) *Il1b*; and (**f**) *Tnfa*. The above genes were measured using RT-PCR and normalized to the housekeeping gene *Actb*. All values are expressed as the mean ± SE (*n* = 6–8). Different letters indicate significant differences (*p* < 0.05, Tukey’s HSD test).

In the liver, hippocampus, and cerebellum, the mRNA expression of *p16^INK4A^* was significantly increased in the CON group compared to that in the Young group (Figure 3a, Figure 4a and Figure 5a, respectively). In contrast, *p16^INK4A^* expression was not elevated in the cerebral cortex (Figure 6a). The expression of other aging markers, *p21* and *p53*, did not differ among the groups in the liver, hippocampus, cerebellum, and cerebral cortex (Figure 3b,c, Figure 4b,c, Figure 5b,c and Figure 6b,c, respectively).

The TEO group showed a tendency to express lower levels of *p16^INK4A^* in the hippocampus than did the CON group (Figure 4a, *p* = 0.0783). The downstream molecules in the p16^INK4A^ pathway, *Cdk4* and *Cdk6*, also showed a tendency to increase in the CON group compared to that in the Young group (*p* = 0.0514 and *p* = 0.0648, respectively, Figure 4g,h). Interestingly, both genes significantly decreased in the TEO group (*p* < 0.05).

Regarding the pro-inflammatory cytokines, the TEO group showed a remarkably lower expression of *Il1b* in the liver and cerebellum (*p* < 0.05, Figure 3e and Figure 5e, respectively) and *Il6* in the hippocampus (Figure 4d) compared to the CON group.

Figure 4i,j depict the mRNA expression of two main transcription factors of NF-κB, *p65* and *p50*, in the hippocampus, where the TEO group exhibited low expression of both genes compared to the CON group (*p* < 0.05).

### 3.6. Aging-Related and Pro-Inflammatory Cytokine Gene Expression in Age-Accelerated NIH-3T3 Cells

**Figure 7:**
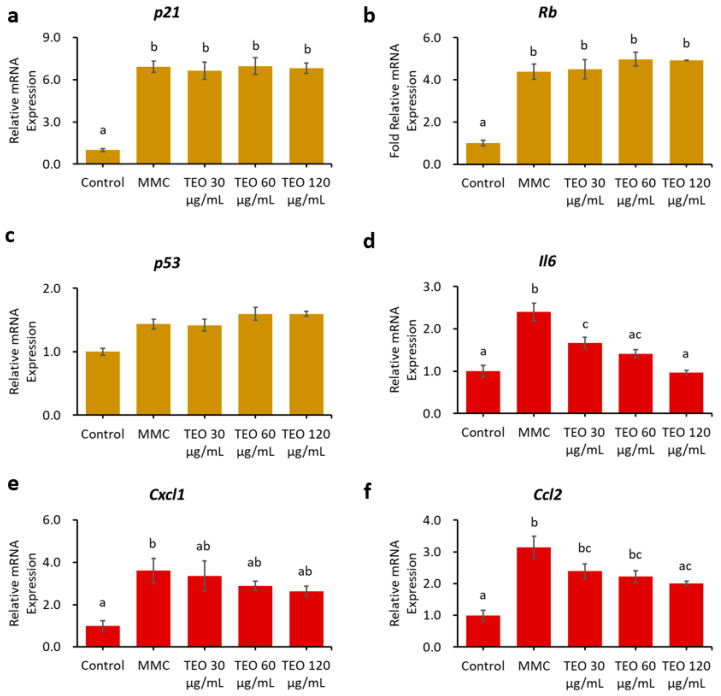
Effects 24-h pretreatment with TEO followed by 24-h co-treatment with TEO and MMC on mRNA expression of aging-related genes: (**a**) *p21*; (**b**) Rb; (**c**) *p53*; (**d**) Pro-inflammatory cytokine *Il6*; Pro-inflammatory chemokines: (**e**) *Cxcl1*; and (**f**) *Ccl2*. All values are expressed as the mean ± SE (*n* = 3). Different letters indicate significant differences (*p* < 0.05, Tukey’s HSD test).

Pretreatment for 24 h with TEO before MMC induction did not ameliorate senescence or inflammation in the NIH/3T3 aging cell model (Figure S4). Conversely, 24-h pretreatment with TEO, followed by co-treatment with TEO and MMC, significantly suppressed the gene expression of pro-inflammatory cytokines and chemokines, as indicated by lower levels of *Il6* and *Ccl2* mRNA expression in a dose-dependent manner (*p* < 0.05, Figure 7d,f).

### 3.7. SA-β-Gal-Positive Cells

**Figure 8:**
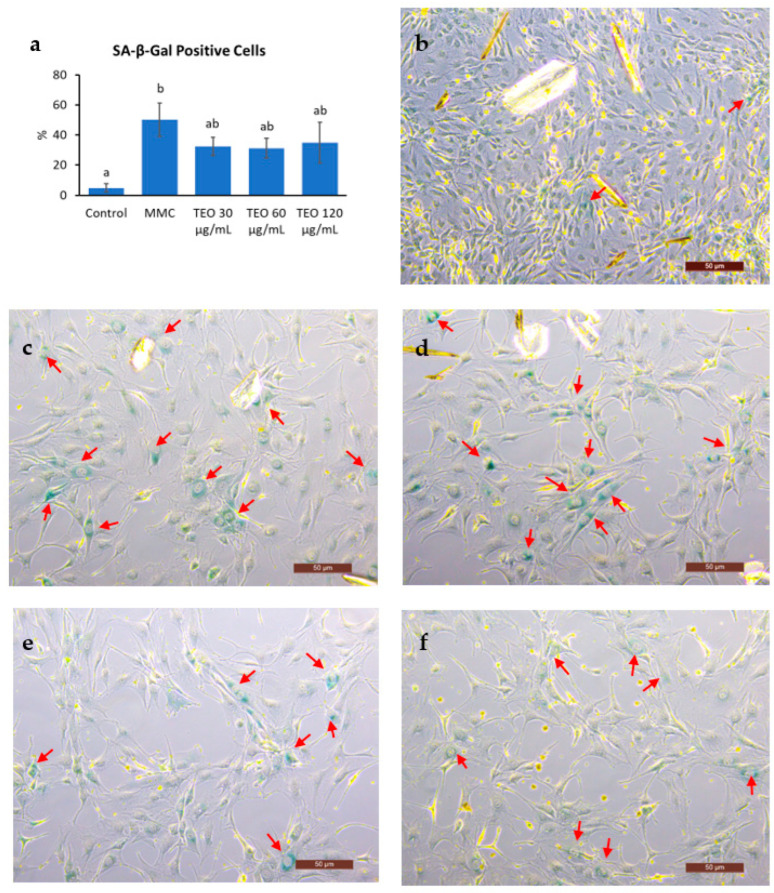
Results of senescence-associated (SA)-β-Galactosidase staining of NIH-3T3 cells. (**a**) Number of SA-β-Gal positive cells. Red arrows show the SA-β-Gal positive cells in (**b**) the control group; (**c**) the group treated with MMC; the groups treated with (**d**) 30 μg/mL TEO, (**e**) 60 μg/mL TEO, (**f**) 120 μg/mL TEO prior to and in conjunction with MMC (pre- and co-treatment). Values in figure (**a**) are expressed as the mean ± SE (*n* = 3). Different letters indicate significant differences (*p* < 0.05; Tukey’s HSD test). Images were captured by a light microscope with 10× magnification. Scale bars indicate 50 μm.

Cell senescence was indicated by a significantly increased number of SA-β-Gal-positive cells after MMC treatment (Figure 8a, *p* < 0.05); however, there was no notable difference between the MMC- and TEO-treated groups. Compared to the control group (Figure 8b), the MMC group showed a larger cell size and smaller number of cells due to DNA damage (Figure 8c–f).

## 4. Discussion

This study is the first to investigate the effects of TEO on aging-associated inflammation in chronologically aged C57BL/6J male mice. While we mainly focused on brain inflammation and telomere attrition, we also examined the changes in body weight, food intake, and liver to provide further insights into how TEO may affect physiological conditions during aging. After 24 weeks of intervention, the results revealed that the TEO diet did not notably affect body weight and food intake compared to the regular diet of the age-matched CON group; however, it lowered retroperitoneal fat content compared to the Young and CON groups (*p* < 0.05, Figure 1d). Retroperitoneal fat is reportedly associated with metabolic syndrome and blood pressure in humans [25]; hence, TEO may be beneficial in preventing metabolic diseases in the elderly. However, further investigation is warranted.

Blood telomere length has been used as a biomarker of longevity [26,27]. Generally, telomere length reduces with age [28,29]; nonetheless, we found that blood telomere length was not significantly different between the Young and CON groups (Figure 2b). Previous studies have indicated that the telomere shortening rate may be different not only between species but also between individuals, owing to genetic factors [27,30,31]. In addition, Khosravaniardakani et al. found that higher body fat mass was also responsible for shorter telomere length [32]. In our study, we observed that all experimental groups had similar total intraperitoneal fat content (Figure 1e).

Interestingly, the TEO group showed a significantly longer telomere length compared to both the Young and CON groups (*p* < 0.05). Telomere metabolism and preservation were found to be correlated to oxidative stress and inflammation [33,34]. Lopez-Otin et al. suggested that reactive oxygen species (ROS) generation increases with age owing to mitochondrial dysfunction [29]. Oxidative stress induces DNA damage, and its accumulation over time accelerates telomere shortening [35]. Thus, antioxidant compounds in food are thought to slow telomere attrition during aging [36]. Curcumin and piperine, for instance, positively affected liver telomere length through their antioxidant and anti-inflammatory activities [37]. Similarly, the antioxidants contained in TEO were predicted to protect telomeres from the elevated ROS generation that usually occurs with aging [29]. As shown in Table 1, the TEO used in this study comprised various antioxidants, mainly monoterpenes, monoterpene alcohols, and phenol derivatives [38]. The main volatile compounds in TEO were thymol (48.96%) and p-cymene (29.15%).

Thymol, a monoterpene phenol, has been widely reported for its strong free radical-scavenging properties [39,40] owing to its phenol group [41]. Also belonging to the monoterpene family is p-cymene, which reduced oxidative stress in a hyperlipidemia rat model [42] and Tween 80-induced oxidative stress in mice [43]. Other important antioxidants contributing to the telomere-protecting effects of TEO are linalool (3.434%), carvacrol (3.409%), and γ-terpinene (3.105%). Of interest, γ-terpinene is a non-phenolic antioxidant that was shown to have synergic antioxidant activity with phenolic antioxidants [44], including thymol. Other chemical compounds in TEO may also synergistically contribute to its antioxidant activities, as Pandur et al. reported that (*E*)-caryophyllene, myrcene, and α-terpinene in TEO also possess antioxidant activities [45].

**Figure 9:**
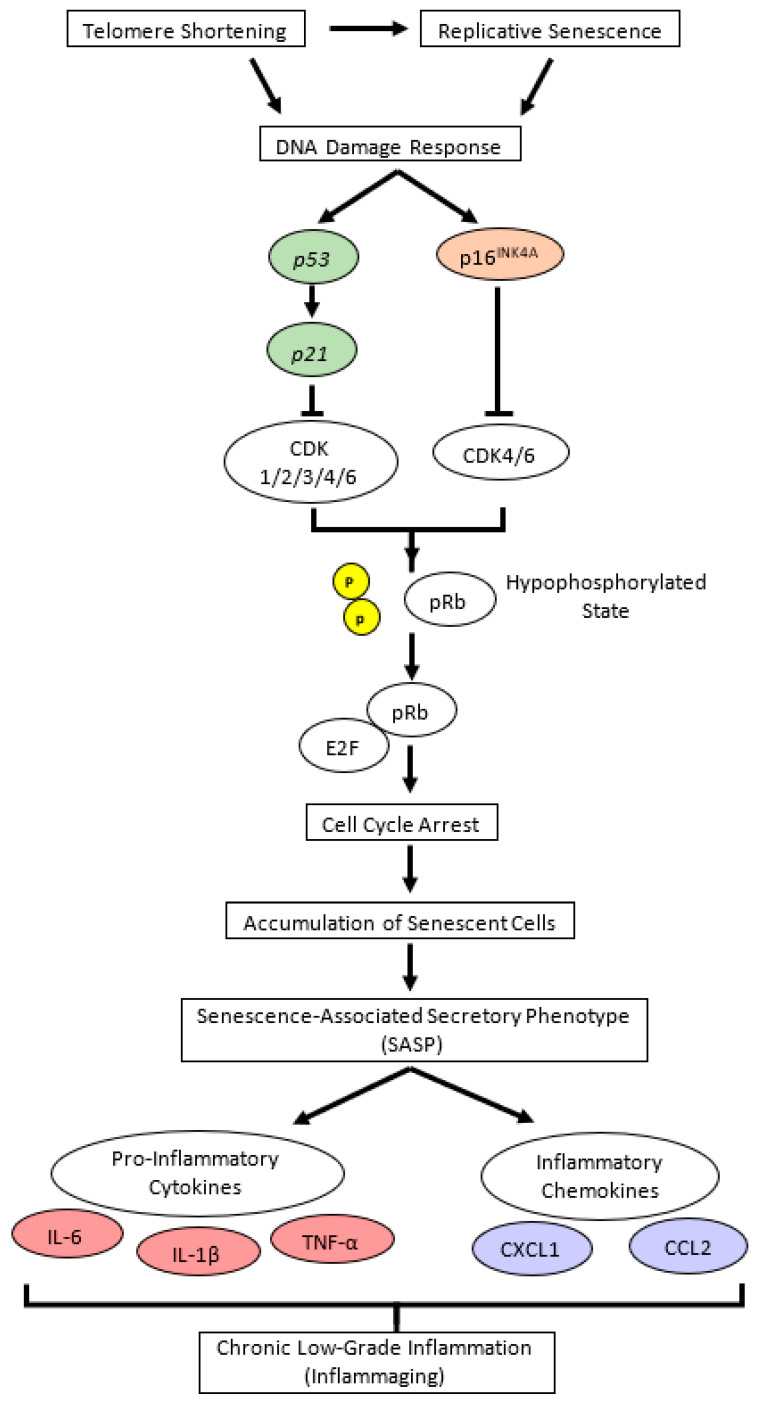
Cellular senescence signaling pathway in chronological (natural) aging. Replicative senescence and telomere shortening trigger DNA damage response, which activates cellular senescence pathways, either through the p16^INK4A^/pRb or p53/p21 pathway. Both p16^INK4A^ and p21 are cyclin-dependent kinase (CDK) inhibitors that bind directly to different CDK proteins; p16^INK4A^ specifically binds to CDK4 and CDK6, whereas p21 allows the binding of wider types of CDK. Further, p16^INK4A^-CDK or p21-CDK complex leads to dephosphorylation of pRb, and under this hypophosphorylated state, pRb binds E2F (a transcription factor for G1 to S phase in cell cycle progression), resulting in cell cycle arrest and senescent cell accumulation. Senescent cells can neither proliferate nor involve in cell repair, yet they remain metabolically active and disrupt normal cells by secreting senescence-associated secretory phenotypes (SASPs), such as pro-inflammatory cytokines and chemokines [2,50]. In age-related cellular senescence, interleukin (IL)-6, IL-1β, tumor necrosis factor (TNF)-α, chemokine C-X-C motif chemokine ligand 1 (CXCL1), and chemokine C-C motif ligand 2 (CCL2) are considered as major pro-inflammatory cytokines and chemokines [5,6]. p in yellow circle illustrates phosphate. CDK, cyclin-dependent kinase; pRb, retinoblastoma protein.

Cellular senescence in aging depends critically on two tumor suppressor pathways: the p53/p21 and p16^INK4A^/pRb pathways [46,47], both of which are interconnected to the inflammatory response (Figure 9). Based on these pathways, *p16^INK4A^*, *p21*, and *p53* were measured as aging markers, whereas *Il6*, *Il1b*, and *Tnfa* were used as pro-inflammatory markers. The CON group had a significantly higher level of *p16^INK4A^* mRNA expression in the liver, hippocampus, and cerebellum (*p* < 0.05) than the Young group but a comparatively unchanged expression of *p21* and *p53*. These data suggested that the p16^INK4A^ pathway was much more dominant in chronological aging than the p53/p21 pathway, which corresponded with a previous report stating that *p16^INK4A^* expression, compared to *p21*, continues to increase during adulthood and is dramatically upregulated in the organs of old animals [48]. A larger fold-change in the expression of *p16^INK4A^* compared to *p21* in most tissues of 22–25-month-old C57BL/6J mice was also previously reported [49].

The increase in *p16^INK4A^* expression was accompanied by increasing mRNA expression of pro-inflammatory cytokines, *Il6*, *Il1b*, or *Tnfa* (*p* < 0.05), depending on the tissue. We predicted that the inflammatory response was most likely a downstream effect of p16^INK4A^ activation during chronological aging (Figure 9).

In the hippocampus, we observed that the mRNA expression of the *p16^INK4A^* gene tended to be lower in the TEO group (*p* = 0.0783, Figure 4a). Activation of the cell senescence pathway is related to prolonged accumulation of DNA mitochondrial damage and oxidative stress [29,51]. Despite our non-significant result, antioxidant compounds may assist the alteration of senescence programming to some extent. This is supported by a previous study demonstrating that two antioxidants, glutathione and melatonin, significantly reduced *p16^INK4A^*, *p21*, and *p53* mRNA expression in senescent adipose tissue-derived mesenchymal stem cells [52]. Other antioxidants, such as quercetin, also demonstrated the same efficacy in downregulating *p16^INK4A^* mRNA expression [8].

Considering the result of *p16^INK4A^* gene expression in the hippocampus, we investigated the effects of TEO on the p16^INK4A^ pathway by measuring the mRNA expression of the downstream molecules, cyclin-dependent kinase *Cdk4* and *Cdk6* (Figure 9). While Cdk is vital for cell cycle progression, p16^INK4A^ is a Cdk-inhibitor; thus, its activation may result in higher mRNA expression of *Cdk4* and *Cdk6* [53] to replenish the required amount for a normal cell cycle. Our data showed that the expression levels of *Cdk4* and *Cdk6* tended to increase in the CON group than in the Young group (*p* = 0.0514 and *p* = 0.0648, respectively) but decreased remarkably in the TEO group (*p* < 0.05). This indicated that TEO intervened in the p16^INK4A^ pathway, which might subsequently prevent the cells from entering a senescent state during spontaneous aging.

The expression of *Il6* was significantly lower in the hippocampus (Figure 4d), which may be linked to the alteration in the p16^INK4A^ pathway, as previous research reported that the suppression of *p16^INK4A^* significantly decreased SASPs [8,54], including IL-6 and CXCL8. However, we also hypothesized that the suppression of *Il6* expression was due to the direct effect of TEO in altering a specific inflammatory pathway, as there was a significant decrease in *Il1b* expression in the liver (*p* < 0.05) and cerebellum (*p* < 0.05) without any decrease in aging markers (Figure 3 and Figure 5, respectively). The ability of thymol to reduce oxidative stress also plays a role in its anti-inflammatory activity [55,56,57]. Moreover, de Santana et al. elucidated the anti-inflammatory effect of p-cymene by modulating the migration of immune cells, especially leukocytes and neutrophils [58].

The anti-inflammatory effects of TEO were also observed in other studies. TEO was reported to modulate the NF-κB signaling pathway to mitigate inflammation [18,45,56]. Xie et al. also demonstrated that p-cymene reduced the production of TNF-α, IL-1β, and IL-6, possibly through NF-κB and MAPK inactivation [59].

Chien et al. found that NF-κB was a major regulator of SASPs, and its activation was accompanied by the accumulation of p16^INK4A^ and p53 proteins, suggesting an inextricable connection between cellular senescence and NF-κB activation [60]. In addition, binding of the transcription factor NF-κB to the IL-6 promoter was enhanced in aged mice compared to young and juvenile mice, indicating that NF-κB regulated *Il6* gene expression in the aged mouse brain [14]. Based on these findings, we measured the gene expression of two primary transcription factors that activate the canonical pathway of NF-κB: *p65* and *p50*. Notably, the TEO diet suppressed their expression (*p* < 0.05, Figure 4i,j).

The hypothesis that TEO may directly affect an inflammatory pathway is supported by our in vitro data. An in vitro experiment was performed to investigate the response of aging cells to TEO treatment. The mouse embryonic fibroblast NIH/3T3 cell line was used, as it has been recommended as a model for studying SASPs owing to its strong SASP phenomenon [61]. Our data showed that 24-h pretreatment with TEO (60 and 120 μg/mL) followed by its co-treatment with MMC remarkably decreased the mRNA expression of the pro-inflammatory cytokine *Il6* (*p* < 0.05) and chemokine *Ccl2* (*p* < 0.05). As aging-related gene expression was not affected by TEO treatment, the number of SA-β-Gal-positive cells was also not significantly different between the MMC- and TEO-treated groups (Figure 8). This result confirmed the anti-inflammatory activity of TEO in an aging cell model.

An important consideration regarding TEO consumption is that it increased *Il1b* expression in the cerebral cortex (*p* < 0.05) compared to that in the Young and CON groups. It is possible that TEO affects SASP factors, including immune response and growth factors, distinctively depending on the tissue type because all tissues experience aging at various rates that are influenced by genetic factors and cell proliferation [62]. As a result, SASP generation and mechanism may be different among tissues. Therefore, so is the effect of TEO. Considering this, a follow-up study on the impact of TEO on immune response and inflammation in brain tissues may be interesting and important to explore in the future.

As aging rates differ among tissues, it may be interesting to study the effects of TEO on various cell types. It has been demonstrated that some plant-derived bioactive compounds may induce senescence in cancer cells [63,64,65]. Gingerenone A, a novel bioactive compound in *Zingiber officinale*, was reported to promote cellular senescence of breast cancer cells (MCF7 and MDA-MB-231) by delaying the G2/M2 phase dan upregulating senescence-associated genes, including *p21* [65]. In addition, subcytotoxic doses of *Z. officinale* extract were reported to induce telomere shortening and cellular senescence in the lung cancer cell line (A549 cells) [63]. Furthermore, the hydroalcoholic extract of *Spartium junceum* L. flowers strongly inhibited the proliferation of B16-F10 murine melanoma cancer cells, while the same concentration did not significantly affect the growth of C2C12 murine myoblasts (non-cancer cells) [64]. This suggests that the impact of plant-derived bioactive compounds on senescence may depend on several factors, including cell types (specifically cancer and non-cancer lines) and treatment dosage. Further investigations of TEO in several cell lines may provide valuable insights into the bioactivity of TEO.

This study is not without limitations. Although we observed notable effects of TEO on the hippocampus, mouse hippocampi were too small to extract sufficient amounts of RNA, DNA, and protein for further analysis to support our current findings. Future studies can address this by using animal models with larger brain sizes, such as rats. In addition, the use of older animals may allow for the observation of stronger SASP phenomenon in naturally aged animals. Finally, it will be crucial to conduct comprehensive investigations on the pharmacokinetics of TEO, including its bioavailability and toxicity, in order to provide more information on the safety of TEO and its potential for broader applications.

## 5. Conclusions

This study is the first to explore the potential effects of TEO on age-related brain inflammation and telomere attrition. TEO, which was previously reported for its potency against neuroinflammation, also showed a notable impact on inflammation in the hippocampus and cerebellum of chronologically aged C57BL/6J male mice. Mice fed the TEO-supplemented diet also showed significantly longer telomeres than those in the other groups. Monoterpenes possessing both antioxidant and anti-inflammatory activities in TEO, particularly thymol and p-cymene, are thought to be important for these effects. Further investigation into the effects of TEO on the immune response and/or a specific inflammatory pathway may be significant to supplement the current findings. Finally, this research is expected to offer a novel anti-aging strategy via dietary antioxidants intervention, such as TEO, to attenuate aging-associated inflammation, which can subsequently support healthy aging.

## Figures and Tables

**Table 1:** Chemical composition of the thyme essential oil (TEO) as analyzed by using gas chromatography/mass spectrometry (GC/MS).

Chemical Compounds	Relative Quantity (%)
α-pinene	1.380
Camphene	0.181
β-pinene	0.010
Myrcene	1.117
α-terpinene	0.109
Limonene	0.746
1,8-cineole	1.149
γ-terpinene	3.105
p-cymene	29.146
Linalool	3.434
Bornyl acetate	1.554
Isobornyl acetate	0.108
β-caryophyllene	0.628
Terpinen-4-ol	0.103
Isoborneol	1.300
α-terpineol	0.721
Borneol	2.113
γ-terpineol	0.404
β-caryophyllene oxide	0.232
Thymol	48.960
Carvacrol	3.409

## Data Availability

All data are available in this article or Supplementary Data. Other inquiries can be directed to the corresponding authors.

## Supplementary Materials

The following supporting information can be downloaded at: https://www.mdpi.com/article/10.3390/antiox12061178/s1. **Table S1**: Diet composition used in the animal experiment. **Table S2**: Mouse primer sequences used for the quantitative real-time polymerase chain reaction (qRT-PCR). **Figure S1**: Mouse brain anatomy [66]. **Figure S2**: General characteristics of mice: body weights and food intake. **Figure S3**: Organ relative weights per 100 g of body weights (BW) in each group after 24 weeks of experimental diet intervention. **Figure S4**: Effects of 24-h pretreatment with TEO on mRNA expression of aging-related genes, pro-inflammatory cytokine, and pro-inflammatory chemokines.
