# Supplementary material for: Effects of Thyme (Thymus vulgaris L.) Essential Oil on Aging-Induced Brain Inflammation and Blood Telomere Attrition in Chronologically Aged C57BL/6J Mice
Source: Antioxidants (Basel). 2023 May 30;12(6):1178. doi: 10.3390/antiox12061178 (PMC10295563; doi:10.3390/antiox12061178)
Supplement: Supplementary file 1 [file antioxidants-12-01178-s001.zip › antioxidants-2397876-supplementary.pdf]

Effects of Thyme (*Thymus vulgaris* L.) Essential Oil on Aging-Induced Brain Inflammation and Blood Telomere Attrition in Chronologically Aged C57BL/6J Mice

Dwina Juliana Warman, Huijuan Jia, and Hisanori Kato

Supplementary Materials

Table S1. Diet composition used in the animal experiment

Ingredients	Amount (% *w*/*w*)	
	**Control**	**TEO**
β-Cornstarch	39.7	39.6
Casein	20.0	20.0
α-Cornstarch	13.2	13.2
Sucrose	10.3	10.3
Soybean oil	7.0	7.0
Fiber	5.0	5.0
Mineral mix（AIN-93G-MX）	3.5	3.5
Vitamin mix（AIN-93G-MX）	1.0	1.0
L-Cystine	0.3	0.3
Thyme Essential Oil	0.0	0.20
**Total**	**100.0**	**100.0**

Table S2. Mouse primer sequences used for the quantitative real-time polymerase chain reaction (qRT-PCR)

Symbol	Gene Name	Accession Number	Sequences (5′ to 3′)
Actb	Beta actin	NM_007393	F: gacggccaggtcatcactat<br>R: cttctgcatcctgtcagcaa
Gapdh	Glyceraldehyde-3-phosphate dehydrogenase	NM_008084	F: tgtgtccgtcgtggatctga<br>R: ttgctgttgaagtcgcaggag
p16^{INK4A}	Cyclin-dependent kinase inhibitor 2A	NM_001040654.1	F: cccaacgccccgaact<br>R: gcagaagagctgctacgtgaa
p21	Cyclin-dependent kinase inhibitor 1A	NM_007669.5	F: cagcctgacagatttctatcactcc<br>R: tcctgacccacagcagaagag
p53	Tumor protein p53	NM_001127233	F: ccgccgacctatccttaccat<br>R: caggcacaaacacgaacctca
Il6	Interleukin 6	NM_031168.2	Liver:<br>F: cttggtccttagccactccttc<br>R: gccagagtccttcagagagataca<br><br>Brain:<br>F: ctggctttgtctttcttgtt<br>R: atttcctctggtcttctgg
Il1b	Interleukin 1 beta	NM_008361.4	F: tcgctcagggtcacaagaaa<br>R: atcagaggcaaggaggaaacac
Tnfa	Tumor necrosis factor alpha	NM_001278601.1	Liver:<br>F: acattcgaggctccagtgaattcgg<br>R: ggcaggtctactttggagtcattgc<br><br>Brain:<br>F: cttgttgcctcctcttttgctta<br>R: ctttatttctctcaatgacccgtag
Rb	Retinoblastoma	XM_036158527	F: aacccagcagtgcgttatct<br>R: ggtgttcgaggtgaaccatt
Cxcl1	C-X-C motif chemokine ligand 1	NM_008176.3	F: actgcacccaaaccgaagtc<br>R: tggggacaccttttagcatctt
Ccl2	Chemokines C–C motif chemokine ligand 2	NM_011333.3	F: gagtagcagcaggtgagtggggcgtta<br>R: cagcaccagcaccagccaactctca

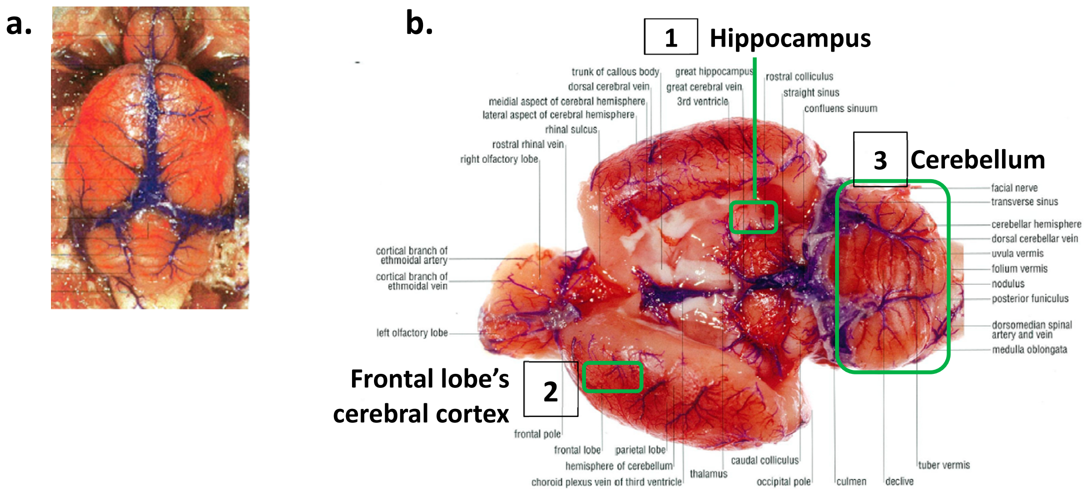

Figure S1. Mouse brain anatomy [66]. (**a**) Top view, (**b**) details of brain parts (bottom view). Parts marked with green box are the location from which 1) hippocampus, 2) cerebral cortex, and 3) cerebellum were collected and analyzed.

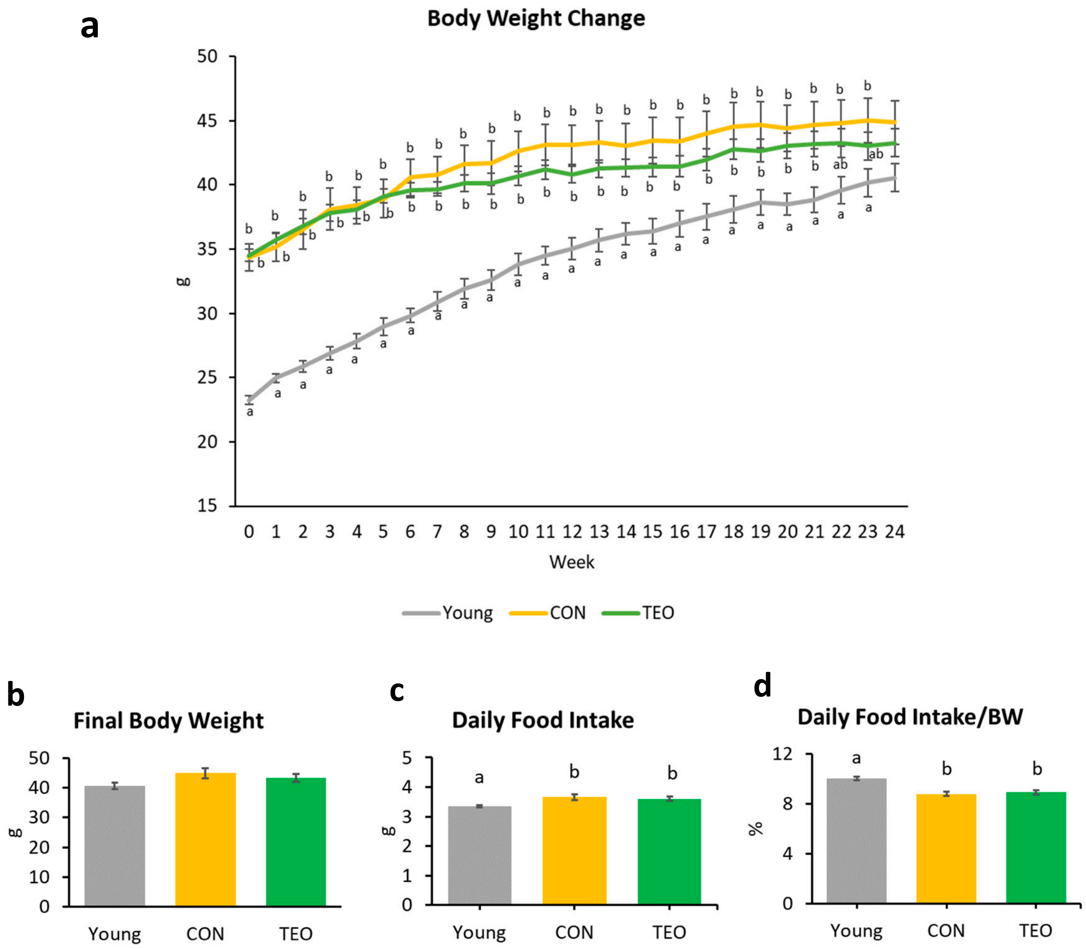

Figure S2. General characteristics of mice. (**a**) Body weight (BW) changes of each experimental group, (**b**) final BW at week 24, (**c**) absolute daily food intake, and (**d**) relative daily food intake per 100 g of body weights. g, gram. All values are expressed as the mean ± SE (n = 7–11). Different letters indicate significant differences (p < 0.05, Tukey's HSD test).

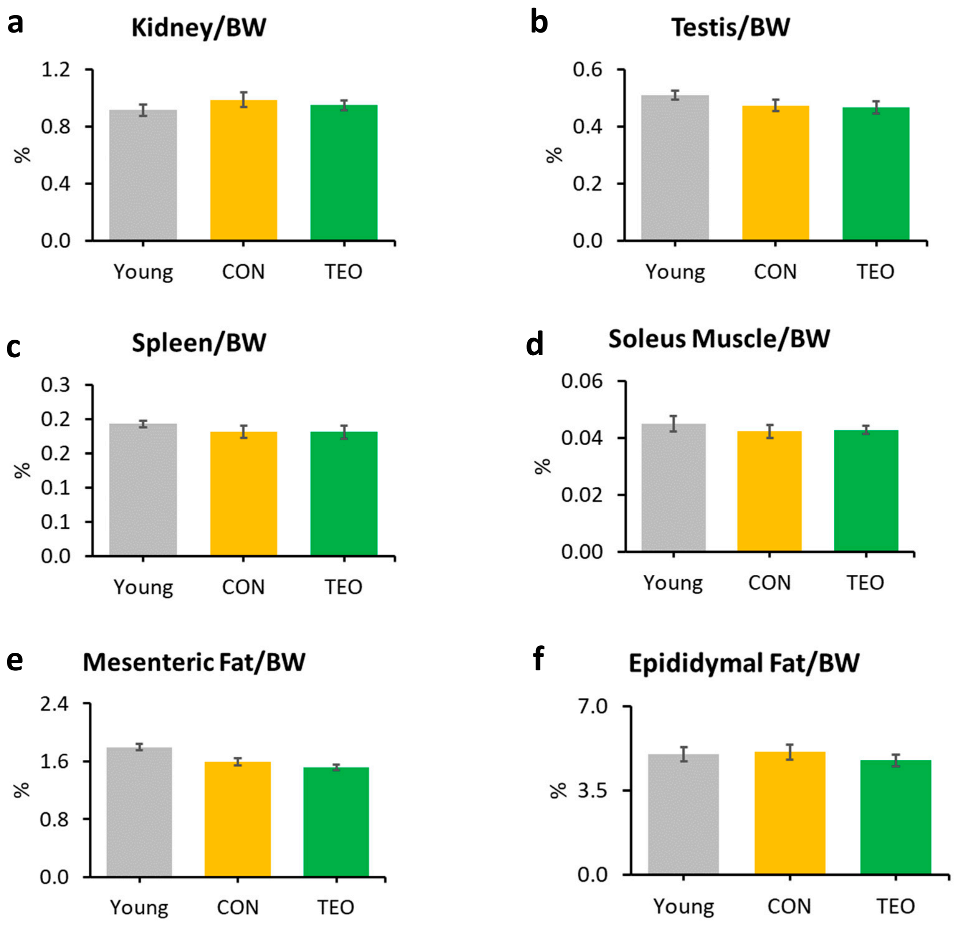

Figure S3. Organ relative weights per 100 g of body weights (BW) in each group after 24 weeks of experimental diet intervention. (**a**) kidney, (**b**) testis, (**c**) spleen, (**d**) soleus muscle, (**e**) mesenteric fat, (**f**) epididymal fat. All values are expressed as the mean ± SE ($n = 7–11$). Different letters indicate significant differences ($p < 0.05$, Tukey's HSD test).

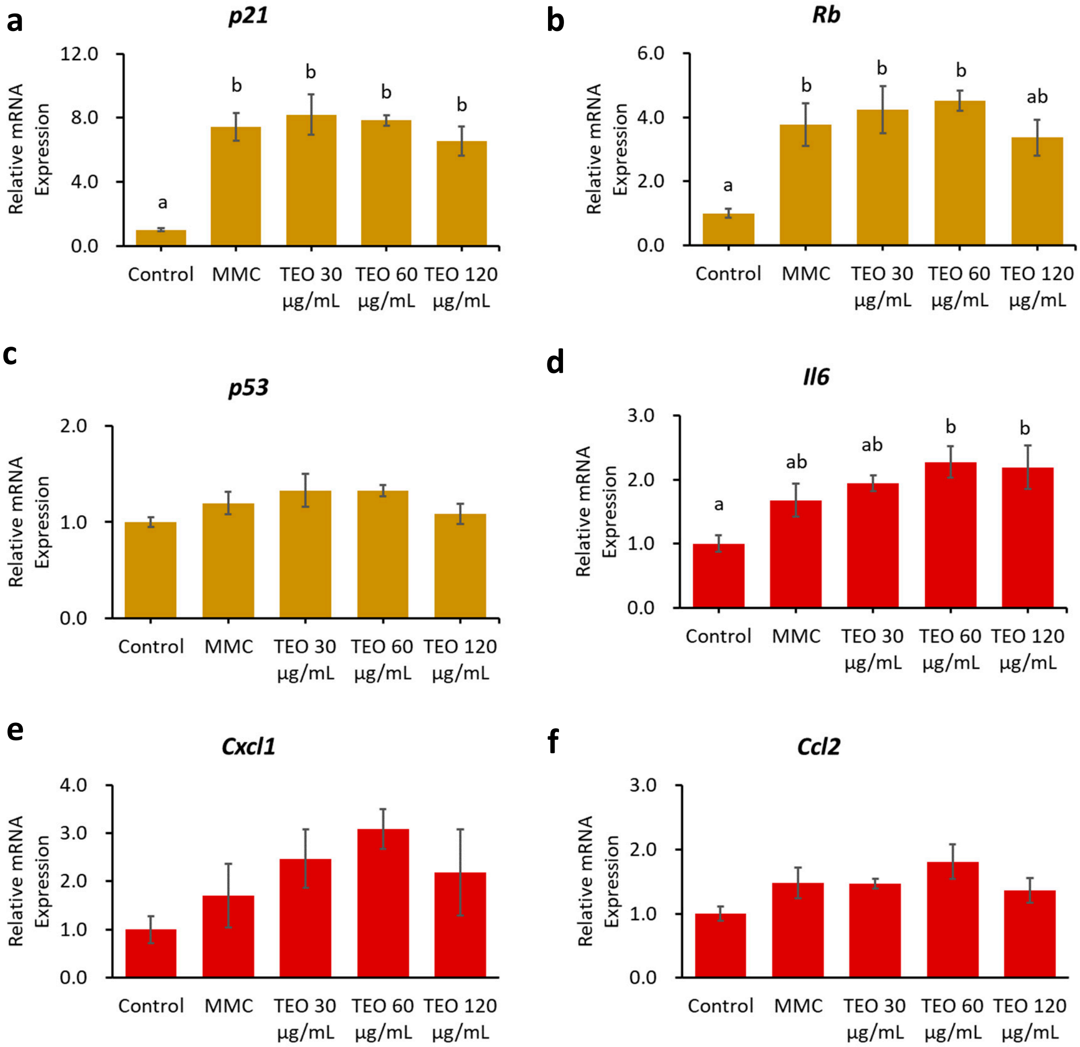

Figure S4. Effects of 24-h pretreatment with TEO on mRNA expression of aging-related genes: (**a**) *p21*, (**b**) *Rb*, (**c**) *p53*; Pro-inflammatory cytokine (**d**) *Il6*; and Pro-inflammatory chemokines: (**e**) *Cxcl1*, (**f**) *Ccl2*. All values are expressed as the mean ± SE ($n = 3$). Different letters indicate significant differences ($p < 0.05$, Tukey's HSD test).
